# Methylene blue as treatment for vasoplegic shock in severe metformin overdose: A case report

**DOI:** 10.1016/j.toxrep.2023.07.005

**Published:** 2023-07-17

**Authors:** Jessica D. Workum, Ala Keyany, Tessa C.C. Jaspers

**Affiliations:** a Department of Intensive Care, Elisabeth-TweeSteden Hospital, Tilburg, the Netherlands; b Department of Electrical Engineering, Eindhoven University of Technology, the Netherlands; c Department of Pharmacy, Elisabeth-TweeSteden Hospital, Tilburg, the Netherlands; d Department of Clinical Pharmacy and Pharmacology, University Medical Center Groningen, the Netherlands

**Keywords:** Metformin toxicity, Methylene blue, Vasoplegic shock, Critically ill

## Abstract

**Introduction:**

Severe metformin overdose can result in life-threatening conditions such as metabolic acidosis with hyperlactatemia and vasoplegic shock. Current treatment guidelines recommend hemodialysis and supportive care. However, this case report presents the use of methylene blue as an additional treatment for severe metformin overdose-induced vasoplegic shock, which is not commonly described in the literature or guidelines.

**Case report:**

A 55-year-old woman presented to the emergency department after ingesting 82.5 g of metformin, resulting in severe metabolic acidosis with hyperlactatemia and refractory vasoplegic shock. Despite continuous hemodialysis and high levels of noradrenalin and vasopressin, the shock persisted. Methylene blue was administered, leading to an immediate and persistent reduction in the noradrenalin dose and rapid shock resolution.

**Discussion:**

This case illustrates the potential use of methylene blue in the treatment of severe metformin overdose. The mechanism for metformin-induced vasoplegia is likely mediated by nitric oxide (NO). Methylene blue has been used to treat NO-mediated vasoplegia in other conditions, such as sepsis and poisoning with beta-blockers and calcium channel blockers, but it is rarely described in metformin toxicity. Methylene blue has a rapid onset of action, with only a few mild side effects. This case report emphasizes the need for clinicians to consider methylene blue as a potential treatment option in cases of refractory vasoplegic shock due to severe metformin overdose.

## Highlights

• Methylene blue leads to rapid shock reversal in severe metformin overdose.
• Methylene blue showed immediate, persistent effects.
• Methylene blue reduces vasopressor needs.
• Methylene blue may contribute to increased lactate clearance.
• Case underscores need for early methylene blue consideration.

## 1 Introduction

Severe metformin overdose is a life-threatening condition that can lead to metabolic acidosis with hyperlactatemia and cardiovascular collapse, including vasoplegic shock. Treatment consists of hemodialysis and supportive care. We present a case of severe vasoplegic shock due to severe metformin toxicity, treated with methylene blue in addition to conventional treatment, which resulted in rapid shock resolution. The use of methylene blue in the treatment of severe metformin overdose has only been described in a few cases and is not described in current guidelines as a treatment option. This case illustrates the potential use of methylene blue in severe metformin overdose.

## 2 Case description

A 55-year-old female (125 kg, body mass index 46 kg/m^2^) presented to the emergency department (ED) after ingestion of 165 tablets of metformin 500 mg (82.5 g, or 660 mg/kg), 20 tablets of acetaminophen 500 mg (10 g, or 80 mg/kg), 38 tablets of simvastatin 40 mg (1520 mg, or 12 mg/kg) and 30 tablets of semaglutide 14 mg (420 mg or 3.4 mg/kg) as a suicide attempt. Immediately after ingestion, she alerted the emergency services herself and presented within 1 h of ingestion. In the ED she was alert and cooperative. Her medical history comprised of earlier suicide attempts with chronic depression and type II diabetes. Her initial vital signs were normal: she had a normal respiratory rate and an oxygen saturation of 95 % without supplemental oxygen, blood pressure was 122/51 mmHg with a normal sinus rhythm of 89/min, and she was alert with a Glasgow Coma Scale of 15. Glucose was mildly elevated (18.4 mmol/L). Her body temperature was 35.7 °C. Initial blood gas analysis showed a pH of 7.19, a pCO2 of 5.6 kPa, bicarbonate of 16 mmol/L, base excess of − 11.8 and lactate levels of 9.5 mmol/L. Liver panel, coagulation and creatine kinase (CK) levels were normal. Serum creatinine was 90 μmol/L. Results are shown in Table 1. Due to the expected severity of the intoxication and the early presentation, she was treated with activated charcoal and immediately admitted to the intensive care unit (ICU) for continuous hemodialysis as the severe lactic acidosis indicated a severe metformin overdose [1].

**Table 1:** Laboratory results during admission.

Measurement	Normal values	Day 0	Day 1	Day 2	Day 3	Day 4
**Hemoglobin (mmol/L)**	8.5–11	8.5	7.2	6.8	6.6	6.3
**Hematocrit**	0.40–0.54	0.43	0.37	0.32	0.32	0.32
**White Blood Cells (10^9/L)^**	4–11	5.3	36.7	24.2	18.7	20.0
**Platelets (10^9/L)^**	150–450	152	244	146	-	85
**Glucose (mmol/L)**	3.9–6.1	18.4	5.6	8.0	10.8	8.1
**Urea (mmol/L)**	2.5–7.8	3.8	0.9	1.3	4.3	4.7
**Creatinine (µmol/L)**	45–90	90	60	51	122	136
**Glomerular Filtration Rate (GFR) (CKD-EPI) (mL/min/1.73 m^2)^**	90–120	62	> 90	> 90	43	38
**Sodium (mmol/L)**	135–145	139	142	137	139	136
**Potassium (mmol/L)**	3.5–5.1	5.2	3.2	4.3	4.5	4.5
**Magnesium (mmol/L)**	0.7–1.0	0.8	0.67	0.71	1.16	1.35
**Phosphate (mmol/L)**	0.8–1.5	1.02	1.17	0.50	1.85	1.51
**Ionized Calcium (mmol/L)**	1.05–1.3	1.01	0.88	0.89	0.87	0.96
**Albumin (g/L)**	35–50	38	-	-	25	-
**Total Bilirubin (µmol/L)**	3–22	11	12	26	78	112
**Alkaline Phosphatase (U/L)**	40–150	107	90	80	139	831
**Gamma-Glutamyl Transferase (GGT) (U/L)**	10–60	40	38	34	71	111
**Aspartate Aminotransferase (ASAT) (U/L)**	10–40	52	224	805	3100	10,518
**Alanine Aminotransferase (ALAT) (U/L)**	10–45	52	99	148	757	4171
**Lactate Dehydrogenase (LDH) (U/L)**	125–220	176	428	782	2799	7896
**pH (arterial)**	7.35–7.45	7.19	7.10	7.43	7.36	7.27
**pO2 (arterial) (kPa)**	11–13	14.3	12.7	9.5	11.3	9.6
**pCO2 (arterial) (kPa)**	4.7–6.0	5.6	6.2	4.9	4.9	6.0
**Bicarbonate (arterial) (mmol/L)**	22–26	16	14.6	24.3	20.5	20.6
**Base Excess (arterial) (mmol/L)**	-2 to + 2	-11.8	-14.6	0.1	-4.4	-6
**Lactate (arterial) (mmol/L)**	0.5–1.6	9.5	25.0	9.2	7.9	4.4

After admission to the ICU, she deteriorated rapidly. She became tachypneic and was intubated for exhaustion. She developed rapid onset shock, which required continuous fluid resuscitation, noradrenalin (rapidly increasing up to 1.2 μg/kg/min) and vasopressin (0.03 IE/min). Hydrocortisone was added because of the refractory nature of the shock. Continuous hemodialysis was initiated within 3 h after presentation. Arterial blood gas and lactate levels were monitored every two hours as a marker for resolution of the metformin overdose. She became hypoglycemic, most likely due to co-ingestion of metformin and semaglutide, for which a continuous 50 % glucose infusion was started. Four hours post-ingestion, approximately three hours after presentation but prior to the initiation of hemodialysis, both acetaminophen and metformin levels were drawn. Acetaminophen levels 4 h after ingestion were 29 mg/L, so treatment with N-acetylcysteine was withheld. Metformin levels were drawn with the intent of retrospective analysis, as the results took one week to complete. Results revealed a level of 622.9 mg/L. However, as these findings were not available during the initial treatment, they had no bearing on medical decision making.

**Fig. 1:**
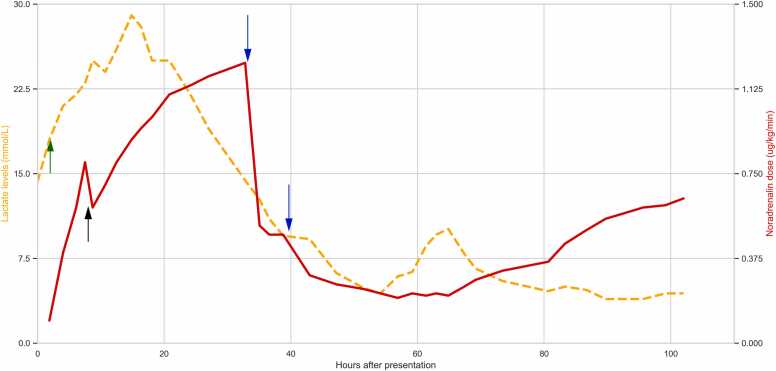
The course of serum lactate (orange dashed line, left y-axis) and noradrenalin dose (red solid line, right y-axis). The green arrow (arrow 1) indicates the initiation of continuous hemodialysis. The black arrow (arrow 2) indicated the addition of vasopressin and hydrocortisone to noradrenalin. The blue arrows (arrows 3 and 4) indicate a bolus of methylene blue 2 mg/kg intravenously. As noradrenalin levels could be rapidly decreased after the first methylene blue injection, the first blue arrow therefore also indicates the start of shock reversal.

Using bedside ultrasonography in conjunction with invasive hemodynamic monitoring using a pulse index continuous cardiac output device (PiCCO), cardiogenic, obstructive, and hypovolemic shock were excluded. Causes of distributive shock other than vasoplegia, such as septic shock and anaphylaxis, were considered unlikely due to the clinical presentation and otherwise normal appearance. As there was no cardiogenic component to the shock, venoarterial extracorporeal membrane oxygenation (va-ECMO) was not considered to be of added value. Therefore, the current condition was considered severe vasoplegic shock due to metformin. As the already high doses of noradrenalin and vasopressin were considered insufficient, we decided to treat the patient with methylene blue. Subsequently, 250 mg of methylene blue (2 mg/kg) was administered intravenously over 5 min. The noradrenalin dose could be reduced from 1.2 μg/kg/min to 0.5 μg/kg/min within 15 min, indicating rapid shock reversal, which was maintained at 0.5 μg/kg/min for 6 h without additional intervention. A second bolus of methylene blue 2 mg/kg was then administered in an attempt to further reduce noradrenalin levels. This allowed the noradrenalin dose to be lowered to 0.25 μg/kg/min (Fig. 1).

The patient remained stable for the next 24 h. Lactate levels decreased from a maximum of 29 mmol/L to 4.4 mmol/L, indicating metformin clearance and improvement of shock. However, the next day, lactate levels began to increase again while still on hemodialysis. She developed severe liver test abnormalities, with alanine aminotransferase (ASAT) of 10518 U/L and aspartate aminotransferase (ALAT) of 4171 U/L, and developed progressive shock again. A computed tomography (CT) scan of both the thorax and abdomen showed extensive necrosis of the liver. As there were no curative options, treatment was switched to palliative care and she passed away. Permission for post-mortem examination was not obtained. However, her next of kin signed informed consent for publication.

## 3 Discussion

We presented a case of severe vasoplegic shock due to metformin toxicity, which was treated with methylene blue in addition to conventional treatment, resulting in rapid shock resolution.

Severe metformin poisoning can lead to metabolic acidosis with hyperlactatemia (metformin associated lactic acidosis, or MALA), glucose derangement (both hyperglycemia and hypoglycemia) and shock. Treatment consists of enhancement of drug elimination via hemodialysis and supportive care. In a scoping review, Juneja et al. summarize the symptomology, clinical interventions and outcomes of 242 patients with metformin poisoning [2]. MALA, defined as lactate levels above 5 mmol/L with concurrent acidosis, was found in 92.6 % of patients and 68.6 % required renal replacement therapy. In patients with acute ingestion, they report a median ingested dose of 42.5 g, mean serum levels of 108.7 mg/L and a mortality of 19.3 %. They did not report any use of methylene blue.

The mechanism of hyperlactatemia in metformin toxicity mainly follows two pathways: the inhibition of mitochondrial glycerol 3-phosphate dehydrogenase (mGPD) and the inhibition of mitochondrial respiratory chain complex 1 (mRCC1) of the electron transport chain [3]. Inhibition of mGPD causes a decrease in gluconeogenesis, which reduces the production of glucose from pyruvate and results in the conversion of pyruvate to lactate. Inhibition of mRCC1 impairs oxidative phosphorylation, leading to mitochondrial dysfunction. This increases the amount of reduced nicotinamide adenine dinucleotide (NADH), which enhances the conversion of pyruvate into lactate. The mechanism for metformin induced vasoplegia is most likely mediated by nitric oxide (NO). Metformin has been shown to increase adenosine monophosphate-activated protein kinase phosphorylation, which activates endothelial nitric oxide synthase (eNOS) and increases NO bioactivity, leading to increased NO levels and subsequent vasodilation [4]. NO-mediated vasoplegia contributes to hyperlactatemia in several ways: first, it leads to shock which causes systemic tissue hypoxia; second, NO itself can cause mitochondrial dysfunction which may increase the production of lactic acid via a mechanism similar to sepsis induced lactic acidosis.

Methylene blue is a commonly used synthetic dye, but is also used in medicine to reverse methemoglobinemia. In toxicology, it is therefore known to reverse the effects of sodium nitrite poisoning [5]. However, methylene blue also reduces NO production, by directly inhibiting NO synthase, but also by binding to the iron heme-moiety of soluble guanylate cyclase, thus competitively blocking the target enzyme of NO [6], [7]. This reduces NO-mediated vasodilation. Therefore, methylene blue has been used in cases where NO-mediated vasoplegia is suspected, such as in sepsis and poisoning with beta-blockers and calcium channel blockers [8], [9].

Methylene blue as rescue therapy for metformin toxicity has only been described in literature in a few case reports. Graham et al. [10] described a case of 44 year old man who ingested 35 g of metformin and developed severe lactic acidosis and shock. He received daily hemodialysis and methylene blue (2 mg/kg bolus with a continuous infusion of 0.25 mg/kg/h for 20 h). He was weaned off vasopressors after 2 days of ICU admission and made a full recovery. Plumb et al. [11] described a case of a 66 year old woman presenting with severe lactic acidosis due to an accidental metformin overdose of unknown quantity, also successfully treated with renal replacement therapy and methylene blue (2 mg/kg loading dose and continuous infusion of 2 mg/kg/h for 12 h). Tallman et al. [12] used va-ECMO as the mainstay of their treatment in addition to conventional treatment, but also describe a beneficial effect of methylene blue on the patient’s blood pressure.

Other than by reducing NO levels, methylene blue may also have a direct positive effect on hyperlactatemia in metformin poisoning. It can act as an alternative electron carrier by accepting electrons from NADH and subsequently delivering them to ubiquinone or cytochrome c, therefore bypassing the electron transport chain impediment at mRCC1, which is impaired in severe metformin poisoning [2]. Therefore, it may also improve MALA. In our patient, this effect could not have been distinguished from the effect of hemodialysis on lactate clearance.

Methylene blue works within minutes and has a maximum effect in 30–60 min after administration. The recommended dose is 1–2 mg/kg intravenously, with a maximum of 7 mg/kg. Approximately 75 % of methylene blue is excreted by the kidneys, either unchanged or as leucomethylene blue. It has a terminal half-life of approximately 25 h [13]. Due to the long half-life of methylene blue, we decided that continuous infusion would not have any benefits over repeated boluses, but would increase the chance of exceeding the recommended dose.

The side effects of methylene blue are mild. They include short-term blue discoloration of the skin, urine and feces, which also occurred in our patient. Other side effects include gastro-intestinal side effects such as nausea and diarrhea. Methylene blue should be administered with caution in patients with glucose-6-phosphate dehydrogenase (G6PD) deficiency, as it can induce hemolytic anemia. In patients with serotonergic co-medication, methylene blue could increase the risk of developing serotonergic syndrome. In both cases, the risk should be weighed against the potential benefits. In doses that exceed the recommended maximum dose of 7 mg/kg, methylene blue can itself induce the formation of methemoglobinemia.

Our patient presented with a severe metformin overdose. She ingested 82.5 g (660 mg/kg), which is double the median dose described in the literature. The metformin level sampled approximately 4 h after ingestion was 622.9 mg/L, which is 6 times the average metformin levels in toxicologic literature [2]. Despite continuous hemodialysis being initiated early, lactate levels continued to rise until 16 h after presentation. Lactate levels served as a treatment efficacy marker: when lactate levels started to decrease, this indicated that metformin levels themselves were also decreasing [14], [15]. We therefore hypothesized that metformin-induced NO production would also decrease. This is why, in contrast to the use of methylene blue in sepsis in which NO production is ongoing, we expected a positive treatment effect of methylene blue in our patient. The effect of methylene blue was immediate and persistent.

Despite being stable for 24 h after the first injection of methylene blue, the patient developed progressive shock again. It is unlikely that this was caused by metformin toxicity, since our patient had been treated with continuous hemodialysis for more than 48 h, given that the half-life for metformin during continuous hemodialysis is approximately 4 h [14]. Therefore, we did not repeat methylene blue as we suspected other causes for the shock. A CT scan showed extensive liver necrosis, which has not been described in metformin toxicity. Considering the known side effects of methylene blue, none of which include liver necrosis or exacerbation of shock, it is unlikely that methylene blue itself contributed to the patient’s worsening condition. Acetaminophen levels 4 h after ingestion were 29 mg/L, which is below the toxic threshold, and liver panel at presentation was normal, ruling out acetaminophen toxicity as a cause. A potential interaction between acetaminophen and simvastatin as a CYP3A4 inducer was considered highly unlikely. As CK levels remained low and no hepatotoxic medication was administered in our ICU, we therefore hypothesize that the severity of the initial shock with high vasopressor doses may have compromised hepatic blood flow, resulting in liver ischemia and subsequent necrosis. This observation further highlights the potential value of methylene blue to reduce vasopressor need in vasoplegic shock. As methylene blue allowed for a rapid reduction in noradrenalin dose in our case, early application could have potentially mitigated the harmful effects of prolonged high-dose vasopressor therapy, such as impaired hepatic blood flow leading to liver necrosis.

## 4 Conclusion

Our patient presented with a metabolic acidosis with hyperlactatemia and a severe vasoplegic shock after a massive metformin overdose. Although scarcely described, methylene blue proved to be a highly effective therapy of vasoplegic shock, with an immediate and persistent effect, allowing a rapid reduction of noradrenalin. As methylene blue has only a few side effects, it is important for clinicians to consider methylene blue when treating patients with refractory shock due to severe metformin overdose.

## Previous presentation

None.

## Funding

None.

## Declaration of Competing Interest

The authors declare that they have no known competing financial interests or personal relationships that could have appeared to influence the work reported in this paper.

## Data Availability

The data that support the findings of this study are available on request from the corresponding author. The data are not publicly available due to privacy or ethical restrictions.
